# IMP-68, a Novel IMP-Type Metallo-β-Lactamase in Imipenem-Susceptible Klebsiella pneumoniae

**DOI:** 10.1128/mSphere.00736-19

**Published:** 2019-10-30

**Authors:** Hiroaki Kubota, Yasunori Suzuki, Rumi Okuno, Yumi Uchitani, Tsukasa Ariyoshi, Nobuyuki Takemura, Fuminori Mihara, Kazuhisa Mezaki, Norio Ohmagari, Mari Matsui, Satowa Suzuki, Tsuyoshi Sekizuka, Makoto Kuroda, Keiko Yokoyama, Kenji Sadamasu

**Affiliations:** a Department of Microbiology, Tokyo Metropolitan Institute of Public Health, Tokyo, Japan; b Department of Hepato-Biliary Pancreatic Surgery, National Center for Global Health and Medicine, Tokyo, Japan; c Microbiology Laboratory, National Center for Global Health and Medicine, Tokyo, Japan; d Disease Control and Prevention Center, National Center for Global Health and Medicine, Tokyo, Japan; e Antimicrobial Resistance Research Center, National Institute of Infectious Diseases, Tokyo, Japan; f Pathogen Genomics Center, National Institute of Infectious Diseases, Tokyo, Japan; JMI Laboratories

**Keywords:** *Enterobacteriaceae*, *Klebsiella*, antibiotic resistance, carbapenems, enzyme kinetics, genome analysis, plasmid-mediated resistance

## Abstract

We recently detected a novel variant of an IMP-type metallo-β-lactamase gene (*bla*_IMP-68_) from meropenem-resistant but imipenem-susceptible Klebsiella pneumoniae TA6363 isolated in Tokyo, Japan. *bla*_IMP-68_ encodes a Ser262Gly point mutant of IMP-11, and transformation experiments showed that *bla*_IMP-68_ increased the MIC of carbapenems in recipient strains, whereas the MIC of imipenem was not greatly increased relative to that of other carbapenems, including meropenem. Kinetics experiments showed that IMP-68 imipenem-hydrolyzing activity was lower than that for other carbapenems, suggesting that the antimicrobial susceptibility profile of TA6363 originated from IMP-68 substrate specificity. Whole-genome sequencing showed that *bla*_IMP-68_ is harbored by the class 1 integron located on the IncL/M plasmid pTMTA63632 (88,953 bp), which was transferable via conjugation. The presence of plasmid-borne *bla*_IMP-68_ is notable, because it conferred antimicrobial resistance to carbapenems, except for imipenem, on *Enterobacteriaceae* and will likely affect treatment plans using antibacterial agents in clinical settings.

**IMPORTANCE** IMP-type metallo-β-lactamases comprise one group of the “Big 5” carbapenemases. Here, a novel *bla*_IMP-68_ gene encoding IMP-68 (harboring a Ser262Gly point mutant of IMP-11) was discovered from meropenem-resistant but imipenem-susceptible Klebsiella pneumoniae TA6363. The Ser262Gly substitution was previously identified as important for substrate specificity according to a study of other IMP variants, including IMP-6. We confirmed that IMP-68 exhibited weaker imipenem-hydrolyzing activity than that for other carbapenems, demonstrating that the antimicrobial susceptibility profile of TA6363 originated from IMP-68 substrate specificity, with this likely to affect treatment strategies using antibacterial agents in clinical settings. Notably, the carbapenem resistance conferred by IMP-68 was undetectable based on the MIC of imipenem as a carbapenem representative, which demonstrates a comparable antimicrobial susceptibility profile to IMP-6-producing *Enterobacteriaceae* that previously spread in Japan due to lack of awareness of its existence.

## Abstract (precis)

IMP-type metallo-β-lactamases comprise one group of the “Big 5” carbapenemases. Here, a novel *bla*_IMP-68_ gene encoding IMP-68 (harboring a Ser262Gly point mutant of IMP-11) was discovered from meropenem-resistant but imipenem-susceptible Klebsiella pneumoniae TA6363. The Ser262Gly substitution was previously identified as important for substrate specificity according to a study of other IMP variants, including IMP-6. We confirmed that IMP-68 exhibited weaker imipenem-hydrolyzing activity than that for other carbapenems, demonstrating that the antimicrobial susceptibility profile of TA6363 originated from IMP-68 substrate specificity, with this likely to affect treatment strategies using antibacterial agents in clinical settings. Notably, the carbapenem resistance conferred by IMP-68 was undetectable based on the MIC of imipenem as a carbapenem representative, which demonstrates a comparable antimicrobial susceptibility profile to IMP-6-producing *Enterobacteriaceae* that previously spread in Japan due to lack of awareness of its existence.

## OBSERVATION

IMP-type metallo-β-lactamases are among the most common families of acquired carbapenemases detected from *Enterobacteriaceae* and have been reported mainly in East Asia, including Japan (1). Although IMP-type metallo-β-lactamases generally hydrolyze carbapenems, this activity on imipenem by several IMP variants, including IMP-6, is weak (2–4). IMP-1 Ser262 is replaced with glycine in these variants, and *Enterobacteriaceae* encoding IMP-6 frequently show susceptibility to imipenem. This feature caused the spread of *bla*_IMP-6_-harboring plasmids in Japan as determined after screening the antibiotic susceptibility of infectious bacteria to imipenem as a representative carbapenem (5–7). Here, we report a novel variant, IMP-68, which is a point mutant of IMP-11 corresponding to the Ser262Gly substitution.

**FIG 1:**
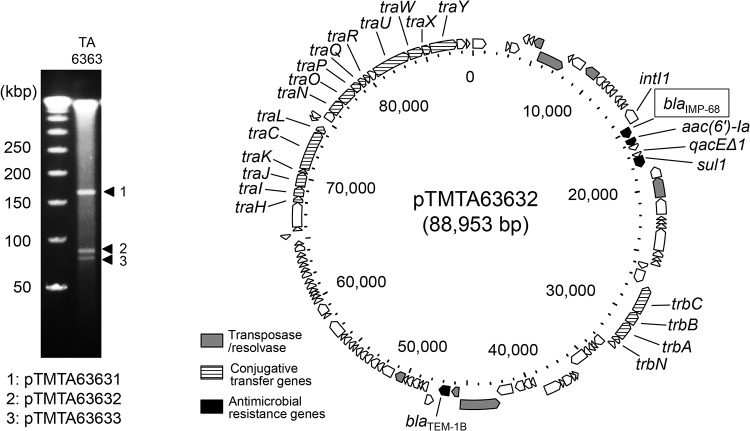
The *bla*_IMP-68_-carrying plasmid pTMTA63632. S1-PFGE pattern of TA6363 showing that three plasmids were carried by TA6363 (left). The circular map of pTMTA63632 (right), which harbored *bla*_IMP-68_, was generated by GView server (https://server.gview.ca/).

Klebsiella pneumoniae TA6363 was isolated from ascites obtained from a hospitalized male patient suffering from peritonitis at the National Center of Global Health and Medicine in 2016. The patient was suspected to be domestically infected by this pathogen, because he had not been abroad from Japan within at least 90 days. This study was approved by the ethics committee of the Tokyo Metropolitan Institute of Public Health. TA6363 was initially determined to be resistant to meropenem by MicroScan walkaway 96 SI (Beckman Coulter, Brea, CA, USA) using the MicroScan Neg Combo EN 1T test card (Beckman Coulter), which employs meropenem as a carbapenem representative. However, this strain was subsequently found to be susceptible to imipenem using the dry strip method using Etest (bioMérieux, La Balme-Les-Grottes, France) (Table 1). TA6363 was positive for a modified carbapenem-inactivation method (8) and found to produce metallo-β-lactamase using the double-disk synergy test involving meropenem and sodium mercaptoacetate (Eiken Chemical, Tokyo, Japan) disks. Pulsed-field gel electrophoresis using an S1-nuclease-digested DNA plug (S1-PFGE) showed that TA6363 carried three plasmids (Fig. 1), with the second larger one (pTMTA63632; ∼90 kbp) harboring the novel *bla*_IMP_ gene according to sequencing plasmids extracted from the S1-PFGE gel (MiSeq; Illumina, San Diego, CA, USA) (9, 10). We found that this *bla*_IMP_ gene encoded a Ser262Gly point mutant of IMP-11 metallo-β-lactamase, and we named this novel variant “*bla*_IMP-68_” (see Fig. S1 in the supplemental material).

**TABLE 1:** MICs of selected antimicrobial agents for the K. pneumoniae TA6363 strain and for the transformants and transconjugants carrying *bla*_IMP-68_ or *bla*_IMP-11_

Antimicrobial agent	MIC (μg/ml) for strain:					
	K. pneumoniae TA6363	E. coli DH5α (pHSG398-*bla*_IMP-68_)	E. coli DH5α (pHSG398-*bla*_IMP-11_)	E. coli DH5α	E. coli J53 (pTMTA63632)	E. coli J53
Ampicillin	>256	16	64	2	>256	4
Piperacillin	>256	1	2	1	>256	2
Ceftazidime	8	64	>256	≤0.06	4	0.125
Cefotaxime	>32	>32	>32	≤0.06	>32	≤0.06
Cefepime	6	4	16	≤0.06	2	≤0.06
Aztreonam	8	<0.06	<0.06	<0.06	0.125	≤0.06
Meropenem	16	32	8	≤0.06	2	≤0.06
Imipenem	0.5	0.5	8	0.125	0.25	0.25
Doripenem	16	4	4	≤0.06	1	≤0.06
Ertapenem	>32	8	1	≤0.06	0.5	≤0.06
Gentamicin	>256	0.125	0.125	0.125	0.25	0.25
Amikacin	8	0.5	0.5	0.5	4	1
Ciprofloxacin	16	≤0.06	≤0.06	≤0.06	≤0.06	≤0.06
Tigecycline	2	0.125	0.125	0.125	0.125	0.125
Polymyxin B	≤0.125	≤0.125	≤0.125	≤0.125	≤0.125	≤0.125

10.1128/mSphere.00736-19.1 FIG S1 Amino acid sequences of IMP variants. The differences in amino acid residues between IMP-1 (or IMP-6) and IMP-68 (or IMP-11) are highlighted in light blue. The residues corresponding to the Ser262Gly substitution are highlighted in magenta. Download FIG S1, TIF file, 1.5 MB. Copyright © 2019 Kubota et al. 2019 Kubota et al. This content is distributed under the terms of the Creative Commons Attribution 4.0 International license.

We tested the influence of IMP-68 on antibiotic resistance to β-lactams by transformation experiments. We cloned the open reading frame of *bla*_IMP-68_ into a chloramphenicol-resistant pHSG398 vector (Takara Bio, Shiga, Japan) at the EcoRI-KpnI site and used it to transform Escherichia coli DH5α cells (Takara Bio). Additionally, we tested *bla*_IMP-11_ for comparison, and conjugation transfer was tested using E. coli J53, as previously described (9). The MICs of β-lactams were determined by the dry strip method using Etest. To determine whether IMP-68 production was detectable by several phenotypic tests other than the modified carbapenem-inactivation method, TA6363 and the E. coli J53 transconjugant were applied to the modified Hodge (11), Carba NP (12), and Blue-Carba (13) tests.

To purify IMP-11 and IMP-68, the nucleotide sequence for the restriction site of the PreScission protease (GE Healthcare, Chicago, IL, USA) was added to the N terminus of *bla*_IMP-11_ and *bla*_IMP-68_ after the signal peptide region (first 19 amino acids) by PCR, followed by cloning into the pETBK vector (BioDynamics, Tokyo, Japan) for expression in E. coli Rosetta II cells (Merck-Millipore, Darmstadt, Germany). His-tagged proteins were purified using a nickel-nitrilotriacetic acid (Ni-NTA) resin (Qiagen, Hilden, Germany) and digested by PreScission protease to remove the His tag.

Kinetics experiments were performed by spectrophotometry (14–16), with initial hydrolysis rates for each β-lactam measured using a Biomate 3 spectrophotometer (Thermo Fisher Scientific, Waltham, MA, USA) at 298 nm for imipenem and meropenem, 260 nm for ceftazidime and cefotaxime, and 235 nm for ampicillin at 24 ± 1°C in 50 mM sodium phosphate buffer (pH 7.0) supplemented with 50 μM ZnCl_2_. *K_m_* and *k*_cat_ values were determined by the Michaelis-Menten equation. Experiments were performed in triplicate.

Table 1 shows the MICs for TA6363 and E. coli transformants. Although TA6363 was resistant to meropenem, doripenem, and ertapenem, the MIC of imipenem for TA6363 was classified as susceptible (S) according to Clinical and Laboratory Standards Institute criteria (12). E. coli transformation by *bla*_IMP-68_ alone significantly increased the MICs of β-lactams, and the substrate specificity of IMP-68 and IMP-11 differed. The lower MIC of imipenem for IMP-68 than that for IMP-11 was similar to the relationship between IMP-6 and IMP-1, where the MIC of imipenem for IMP-6 is lower than that for IMP-1 (2). Furthermore, the *bla*_IMP-68_-carrying native plasmid pTMTA63632 was transferred by conjugation from TA6363 to E. coli J53, resulting in enhanced antibiotic resistance via pTMTA63632. TA6363 and the transconjugant were positive for all the above-mentioned tested phenotypic methods. Despite the use of imipenem, the Carba NP and Blue-Carba tests were sensitive enough to detect IMP-68 production.

Several differences in substrate specificity between IMP-11 and IMP-68 identified during antibiotic susceptibility testing were consistent with the results of kinetics experiments (Table 2). The *k*_cat_/*K_m_* values of IMP-68 against imipenem were lower than those of IMP-11, whereas IMP-68 exhibited higher meropenem-hydrolyzing activity. Additionally, the *k*_cat_/*K_m_* values of IMP-68 against ceftazidime were lower than those of IMP-11. These differences in substrate specificity between IMP-68 and IMP-11 correlated with those between IMP-6 and IMP-1 (2).

**TABLE 2:** Kinetic parameters of IMP-11 and IMP-68

Antimicrobial agent	IMP-68			IMP-11		
	*K_m_* (μM)^*a*^	*k*_cat_ (s^−1^)^*a*^	*k*_cat_/*K_m_* (μM^−1^ s^−1^)	*K_m_* (μM)^*a*^	*k*_cat_ (s^−1^)^*a*^	*k*_cat_/*K_m_* (μM^−1^ s^−1^)
Ampicillin	465 ± 200	1.7 ± 0.44	0.0037	830 ± 248	15.0 ± 1.9	0.018
Ceftazidime	326 ± 131	3.9 ± 2.1	0.012	232 ± 18.9	8.8 ± 3.7	0.038
Cefotaxime	10.3 ± 2.3	25.7 ± 1.3	2.5	11.8 ± 2.2	9.0 ± 1.6	0.84
Meropenem	10.1 ± 0.94	9.7 ± 1.7	0.89	14.8 ± 2.8	5.5 ± 0.50	0.37
Imipenem	347 ± 52.2	36.7 ± 7.8	0.11	41 ± 18.4	21.9 ± 4.7	0.54

a Presented as the mean ± standard deviation.

Additionally, genomic DNA extracted from TA6363 was sequenced on MiSeq and MinION platforms (Oxford Nanopore, Oxford, United Kingdom), and the obtained reads were assembled by Unicycler (v.0.4.7) (17). Genes were predicted and annotated using Prokka (v.1.11) (18), NCBI BLAST (https://blast.ncbi.nlm.nih.gov/Blast.cgi), and ResFinder (v.3.1) (19). The Inc types of the plasmids were determined by PlasmidFinder (v.2.0) (20), with the threshold of nucleotide coverage and identity used for Inc-type identification established at 96% and 98%, respectively (21).

We obtained four circular sequences (Table S1) corresponding to a chromosome and three plasmids (Fig. 1 and Fig. S2). TA6363 was classified as ST268, and pTMTA63632 was determined to be an IncL/M plasmid. Chromosomal mutations corresponding to antimicrobial resistance to carbapenems (*ompK36*, *ompK37*, and *acrR*) and fluoroquinolones (*gyrA* and *parC*) were not found (Table S1). Class 1-integron-harboring *bla*_IMP-68_ in pTMTA63632 (Fig. 1), which was assigned to In1702 in the INTEGRALL database (22), was similar to previously reported IMP genes harboring class 1 integrons, as the gene cassettes were located between *intI1* and *qacEΔ1-sul1* (10, 23, 24). Specifically, In1702 was compared with the class 1 integrons harbored by pNUH14_ECL028_1 and pIMP-A2015-49 plasmids previously found in Japan (24), which carried IMP-1 and IMP-11, respectively (Fig. S3). The inclusion of conjugation transfer genes by pTMTA63632 was consistent with the conjugation experiment performed using E. coli J53 (Table 1). The higher MICs of ampicillin and piperacillin for the transconjugant than for *bla*_IMP-68_ alone (Table 1) likely originated from the effect of *bla*_TEM-1_ in pTMTA63632. TA6363 was resistant to both amikacin and gentamicin, whereas the transconjugant was resistant only to amikacin. This difference was attributable to the carriage of aminoglycoside resistance genes by pTMTA63632 [*aac(6′)-Ia*] and pTMTA63633 [*aac(3)*-*IId*, *aph(3′)*-*Ib*, and *aph(6)*-*Id*] (Table S1) (25); namely, pTMTA63632 conferred antimicrobial resistance to amikacin, whereas pTMTA63633 was additionally necessary for the gentamicin resistance.

10.1128/mSphere.00736-19.2 FIG S2 Circular maps of plasmids pTMTA63631 and pTMTA63633. These maps were generated by GView server (https://server.gview.ca/). Conjugative transfer genes were not detected in these plasmids. Download FIG S2, TIF file, 1.7 MB. Copyright © 2019 Kubota et al. 2019 Kubota et al. This content is distributed under the terms of the Creative Commons Attribution 4.0 International license.

10.1128/mSphere.00736-19.3 FIG S3 Comparison of *bla*_IMP_-harboring integrons isolated in Japan. The Artemis comparison tool was used to compare In1702 in pTMTA63632 with previously reported *bla*_IMP-1_- and *bla*_IMP-11_-harboring integrons. These integrons were found in plasmids pNUH_ECL028_1 (for *bla*_IMP-1_) and pTMP-A2015-49 (for *bla*_IMP-11_) carried by bacteria isolated in Japan in 2014 and 2015, respectively. Because the original data for pNUH_ECL028_1 were submitted to a database without gene annotation, we predicted and annotated its genes by the same method with pTMTA63632 in this study (see text). Similar regions (BLAST score of >300 and identity of >85%) are shown by magenta lines. Downstream sequence of pTMP-A2015-49 was not registered in the database and thus could not be compared with pTMTA63632. The open reading frames colored in light blue and gray indicate antimicrobial resistance genes and transposases, respectively. Download FIG S3, TIF file, 1.0 MB. Copyright © 2019 Kubota et al. 2019 Kubota et al. This content is distributed under the terms of the Creative Commons Attribution 4.0 International license.

10.1128/mSphere.00736-19.4 TABLE S1 Profiles of the TA6363 chromosome and plasmids. Download Table S1, DOCX file, 0.02 MB. Copyright © 2019 Kubota et al. 2019 Kubota et al. This content is distributed under the terms of the Creative Commons Attribution 4.0 International license.

In conclusion, IMP-68 should be noted as a novel carbapenemase that does not sufficiently confer resistance to imipenem on *Enterobacteriaceae* due to the Ser262Gly substitution from IMP-11. IMP-68 production would have been missed if the MIC of imipenem had been used to investigate the carbapenemase-producing *Enterobacteriaceae*. The diversity of substrate specificity among IMP-type enzymes might affect treatment plans using antibacterial agents in clinical settings.

### Data availability.

We deposited the *bla*_IMP-68_ sequence in GenBank (MF669572) and the whole-genome sequence of TA6363 containing chromosome (AP019665), pTMTA63631 (AP019666), pTMTA63632 (AP019667), and pTMTA63633 (AP019668) in the DNA Data Bank of Japan (DDBJ).
