# Supplementary figures and images for: IMP-68, a Novel IMP-Type Metallo-β-Lactamase in Imipenem-Susceptible Klebsiella pneumoniae
Source: mSphere. 2019 Oct 30;4(5):e00736-19. doi: 10.1128/mSphere.00736-19 (PMC6821933; doi:10.1128/mSphere.00736-19)

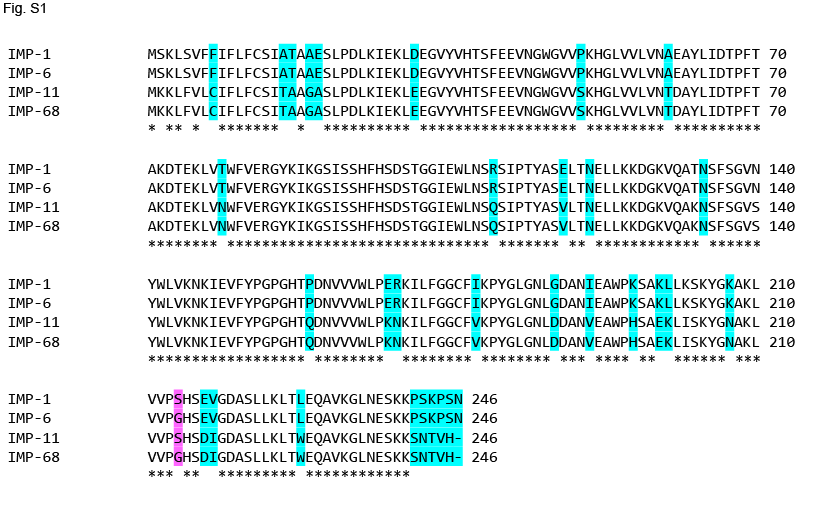

Supplement: FIG S1 [file mSphere.00736-19-sf001.tif]

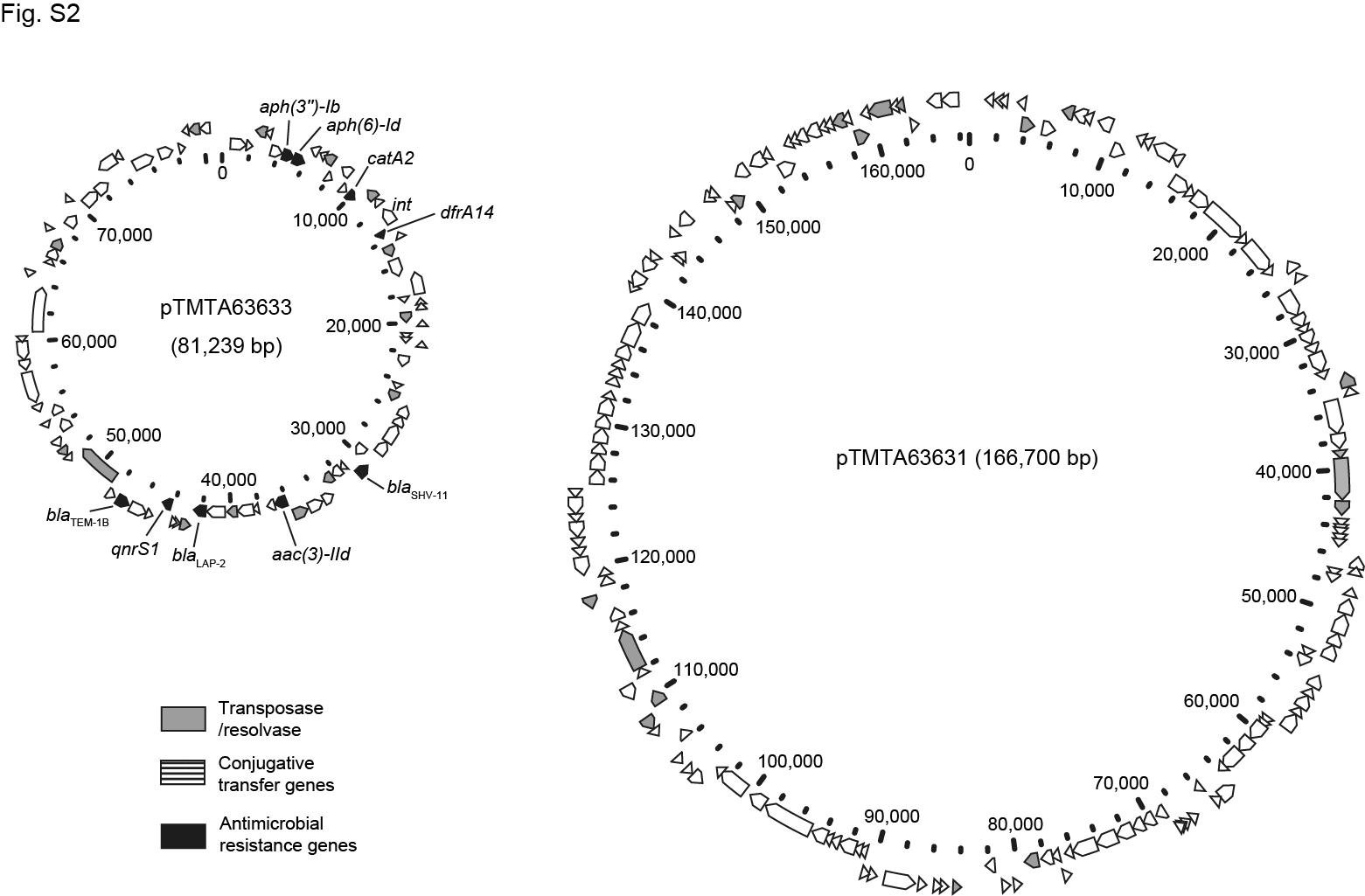

Supplement: FIG S2 [file mSphere.00736-19-sf002.tif]

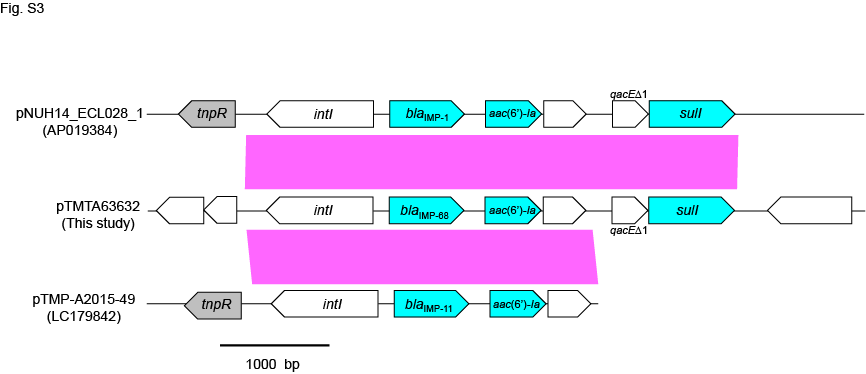

Supplement: FIG S3 [file mSphere.00736-19-sf003.tif]
