# Supplementary material for: IMP-68, a Novel IMP-Type Metallo-β-Lactamase in Imipenem-Susceptible Klebsiella pneumoniae
Source: mSphere. 2019 Oct 30;4(5):e00736-19. doi: 10.1128/mSphere.00736-19 (PMC6821933; doi:10.1128/mSphere.00736-19)
Supplement: TABLE S1 [file mSphere.00736-19-st001.docx]

**TABLE S1** Profiles of the TA6363 chromosome and plasmids

| Chromosome  /Plasmid | Length (bp) | Number of genes | Inc type | Acquired antimicrobial-resistance genes | Chromosomal mutations | | | | |
| --- | --- | --- | --- | --- | --- | --- | --- | --- | --- |
|  |  |  |  |  | *ompK35* | *ompK36* | *acrR* | *gyrA* | *parC* |
| Chromosome | 5,318,671 | 4,910 | N/A | *oqxB*, *oqxA*, *bla*_SHV-13_, *fosA5* | Not found | Not found | Not found | Not found | Not found |
| Plasmid  pTMTA63631 | 166,700 | 171 | Not identified^a^ | Not found | N/A | N/A | N/A | N/A | N/A |
| Plasmid  pTMTA63632 | 88,953 | 114 | IncL/M(pMU407) | *aac*(6')-*Ia*, *bla*_IMP-68_,  *bla*_TEM-1B_, *sul1* | N/A | N/A | N/A | N/A | N/A |
| Plasmid  pTMTA63633 | 81,239 | 92 | IncFIA(HI1), IncR | *dfrA14*, *bla*_SHV-13_, *sul2*, *aac*(3)-*IId*, *aph*(3'')-*Ib*, *qnrS1*, *bla*_TEM-1B_, *aph*(6)-*Id*, *catA2* | N/A | N/A | N/A | N/A | N/A |

^a^ pTMTA63631 exhibited 100% and 91.3% nucleotide coverage and identity, respectively, with IncFIB(K).
